# Effect of major lifestyle risk factors, independent and jointly, on life expectancy with and without cardiovascular disease: results from the Consortium on Health and Ageing Network of Cohorts in Europe and the United States (CHANCES)

**DOI:** 10.1007/s10654-015-0112-8

**Published:** 2016-01-18

**Authors:** Mark G. O’Doherty, Karen Cairns, Vikki O’Neill, Felicity Lamrock, Torben Jørgensen, Hermann Brenner, Ben Schöttker, Tom Wilsgaard, Galatios Siganos, Kari Kuulasmaa, Paolo Boffetta, Antonia Trichopoulou, Frank Kee

**Affiliations:** UKCRC Centre of Excellence for Public Health for Northern Ireland, Queens University Belfast, Belfast, BT12 6BA Northern Ireland, UK; Centre for Statistical Science and Operational Research (CenSSOR), Queen’s University Belfast, Belfast, BT7 1NN Northern Ireland, UK; Research Centre for Prevention and Health, Glostrup University Hospital, Glostrup, Denmark; Institute of Public Health, University of Copenhagen, Copenhagen, Denmark; Faculty of Medicine, University of Aalborg, Aalborg, Denmark; Division of Clinical Epidemiology and Aging Research, German Cancer Research Center (DKFZ), Im Neuenheimer Feld 581, 69120 Heidelberg, Germany; Department of Community Medicine, University of Tromsø, 9037 Tromsø, Norway; Department of Health, National Institute for Health and Welfare (THL), 00271 Helsinki, Finland; The Tisch Cancer Institute and Institute for Translational Epidemiology, Mount Sinai School of Medicine, New York, NY 10029 USA; Hellenic Health Foundation, Kaisareias 13 & Alexandroupoleos str., 115 27 Athens, Greece; Department of Hygiene, Epidemiology and Medical Statistics, Medical School, University of Athens, Mikras Asias 75 st, 115 27 Athens, Greece

**Keywords:** CHANCES, Ageing, Smoking, Obesity, Cardiovascular disease, Mortality

## Abstract

Seldom have studies taken account of changes in lifestyle habits in the elderly, or investigated their impact on disease-free life expectancy (LE) and LE with cardiovascular disease (CVD). Using data on subjects aged 50+ years from three European cohorts (RCPH, ESTHER and Tromsø), we used multi-state Markov models to calculate the independent and joint effects of smoking, physical activity, obesity and alcohol consumption on LE with and without CVD. Men and women aged 50 years who have a favourable lifestyle (overweight but not obese, light/moderate drinker, non-smoker and participates in vigorous physical activity) lived between 7.4 (in Tromsø men) and 15.7 (in ESTHER women) years longer than those with an unfavourable lifestyle (overweight but not obese, light/moderate drinker, smoker and does not participate in physical activity). The greater part of the extra life years was in terms of “disease-free” years, though a healthy lifestyle was also associated with extra years lived after a CVD event. There are sizeable benefits to LE without CVD and also for survival after CVD onset when people favour a lifestyle characterized by salutary behaviours. Remaining a non-smoker yielded the greatest extra years in overall LE, when compared to the effects of routinely taking physical activity, being overweight but not obese, and drinking in moderation. The majority of the overall LE benefit is in disease free years. Therefore, it is important for policy makers and the public to know that prevention through maintaining a favourable lifestyle is “never too late”.

**Electronic supplementary material:**

The online version of this article (doi:10.1007/s10654-015-0112-8) contains supplementary material, which is available to authorized users.

## Introduction

The World Health Organization estimates that 17.3 million people died from cardiovascular diseases (CVD) in 2008, representing 30 % of all deaths [1]. Of these deaths, 7.3 million resulted from coronary heart disease (CHD) and 6.2 million from stroke [1]. By 2030, almost 23.6 million people will die from CVD, mainly from CHD and stroke, and these are projected to remain the single leading causes of death [2].

Across the European region there are significant variations in total life expectancy (LE_tot_) and in the proportion of life expectancy (LE) lived without significant self-reported disease or disability [3]. As our LE_tot_ increases, whether or not the number of years lived with morbidity in old age will be compressed [4] is a subject of some debate. Some risk factors in older age may affect incidence and mortality in divergent ways. Obesity for example increases incidence risk but apparently has either no effect or may be protective among those who have experienced a cardiovascular event [5]. While there has been some decline in the prevalence of smoking, current trends in physical activity, obesity and alcohol consumption are adverse. Few studies, however, have investigated the independent and joint effects of these lifestyle factors on LE_tot_ and LE free of CVD [6]. Previous studies suggest that not smoking [7], moderate/high levels of physical activity [8], and normal weight [9] each are associated with a longer LE free of CVD and LE_tot_, but to a different extent. Several studies also show a protective effect of light/moderate and regular alcohol consumption on total mortality and CVD mortality [10–12]. The effects on the number of years lived with CVD also appeared to vary between these behaviours [7–9].

However, it remains unclear to what extent these results reflect real differences in the risk factors’ effects, and even those studies that have analysed all three behaviours have not examined their joint effects [6]. In addition, previous studies have seldom accounted for changes in risk factors and there has been a resurgent interest in such issues given the possibility that some risk factors may have effects that are additional and independent from those of a single baseline assessment [13].

Finally, few studies have looked at CVD outcomes in countries where lifestyle habits are known to contrast markedly, which may further help elucidate why outcomes vary between countries [3].

CHANCES is the Consortium on Health and Ageing: Network of Cohorts in Europe and the United States and as such includes a large number of cohorts from all over Europe and the United States [14]. We use data from well characterised CHANCES cohorts (ESTHER, Germany; RCPH, Denmark; and Tromsø, Norway) which have the appropriate repeated measures (of risk factor covariates) available to analyse how LE with and without CVD is related to the independent and joint effects of smoking, physical activity, obesity and alcohol consumption, in populations aged 50+ years from different countries.

## Methods

### Study design and study population

The aim of the CHANCES project is to combine and integrate prospective cohort studies in order to produce evidence on ageing-related health characteristics and determinants [14]. The same analysis script, including the harmonised endpoints and other variables as outlined within CHANCES were applied in all cohorts assuring a high level of comparability. Due to differences in follow up times, the number of re-contacts, and length of time between each re-contact, individual cohort analysis was considered best suited to our purpose rather than attempting an individual participant meta-analysis. The procedures followed in all of the cohorts were in accordance with the ethical standards of the responsible institutional or regional committee on human research. Written informed consent was obtained from all participants.

RCPH, Denmark [15]—Participants at baseline (n = 3785) were excluded if they had prevalent CVD (n = 90) or were <50 years (n = 1936). The sample size consisted of 1759 individuals at baseline (1982–1984); 1377 at the first recontact (1987–1988; R1) and 1120 at the second recontact (1993–1994; R2).

ESTHER, Germany [16]—Participants at baseline (n = 9949) were excluded if they had prevalent CVD (n = 1209), or were <50 years (n = 18). The overall sample size at baseline (2000–2002) consisted of 8482 individuals; 7329 individuals at R2 (2005–2007) and 6242 individuals at R3 (2008–2010).

Tromsø, Norway [17]—For this study, Tromsø surveys T4 and T5 were included. Participants at T4 baseline (n = 10,252) were excluded if they had prevalent CVD (n = 1073). The sample size consisted of 9179 individuals at baseline (1994–1995) and 5211 individuals at T5 (2001). All participants were ≥50 years at baseline.

### Exposures and covariates

At baseline, height and weight was assessed and documented in all cohorts. Similar procedures were adhered to at subsequent re-contacts, except ESTHER which collected self-reported height and weight; those who had a home-visit at R3 (~45 %) had these anthropometric measurements documented. Age, sex, smoking status (never, former, current), alcohol intake [abstainer (0 g daily); light/moderate = men (>0 g and <60 g daily), women (>0 g and <40 g daily); heavy = men (≥60 g daily), women (≥40 g daily)], physical activity (any vigorous activity at least once per week to cause increased breathing/sweating, yes/no), hypertension (based on measured blood pressure and hypertensive drug treatment use) and total/HDL cholesterol ratio were available in all cohorts. A variable based on a combination of self-reported hypertension and hypertensive drug treatment use was employed as a proxy for R2 in ESTHER as blood pressure was not measured at this recontact. Prevalent diabetes was also available, but was documented in ESTHER and self-reported in RCPH and Tromsø. All variables used in the analyses from different cohorts were harmonised according to pre-agreed CHANCES data harmonisation rules [18].

### Outcomes

All cohorts obtained the exact date of death from an official death register. Follow-up of fatal and nonfatal CVD [acute coronary event or stroke (type unspecified)] used similar techniques, including responses to follow-up questionnaires, hospital discharge registers and general practitioner or independent endpoint committee confirmation. More detailed descriptions of the cohorts, exposures, covariates and outcomes are available online [18].

### Statistical methods

**Fig. 1:**
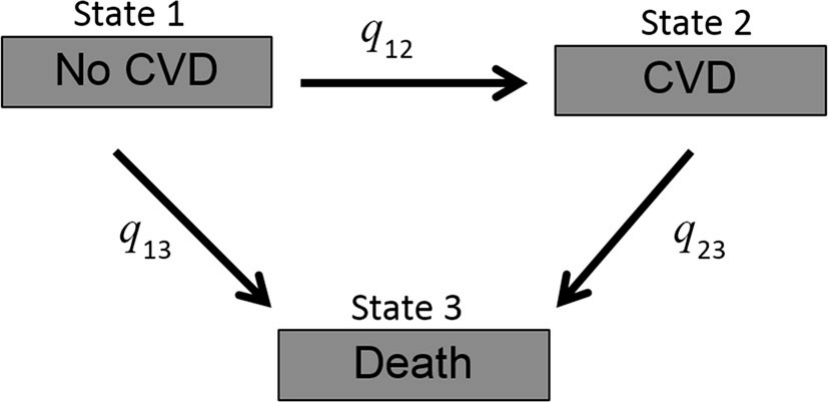
Multistate Markov model used with individuals being in one of three possible states at time *t*: *X*_*t*_ = 1 (free of CVD), *X*_*t*_ = 2 (nonfatal CVD) or *X*_*t*_ = 3 (all-cause death)

A multi-state Markov model was employed [19], being a useful way of describing a process in which an individual moves between states in continuous time. Here a non-recoverable illness-death model was constructed (see Fig. 1, with individuals starting free of CVD in state 1 at time *t*, and moving to either a nonfatal CVD event in state 2 or death of any cause at state 3—competing risk), in order to assess associations between each of the major lifestyle behaviours (smoking, physical activity, BMI and alcohol consumption) and LE with and without CVD. Individuals who suffer a fatal CVD event (or die from any cause) move directly from state 1 to state 3 without first moving to state 2, while those who have a nonfatal CVD event move from state 1 to state 2 and then either stay in state 2 or move to state 3 if they should die from any cause at a later follow-up point. When using the repeated measures of covariates in the Markov model, the most recent available value for each measurement was used in the analysis when an event occurred.

The parameters of such a three state model were estimated through use of the R msm package [20] with the instantaneous risk of moving from state *i* to state *j* influenced by the characteristics of individuals (either time-dependent or constant covariates) in a proportional hazards fashion. LE was calculated following the techniques of the R ELECT package [21].

A secondary joint analysis was performed, where men and women were grouped separately into two lifestyle categories: “favourable” (those who are overweight but not obese, light/moderate drinkers, and are non-smokers and participate in vigorous physical activity); versus “unfavourable” (those who are overweight but not obese, light/moderate drinkers, and are smokers and do not participate in vigorous physical activity) [Because of small cell counts within joint categories, and the consequent non-convergence of the MSM models, it was not possible to create “lifestyle” stereotypes in which all four risk factors varied.] The same statistical techniques as outlined above were used for this joint analysis.

A detailed description of the statistical methods employed can be found in the Supplementary Material, Online Resource.

## Results

Table 1 shows the baseline characteristics of all three cohorts. With the exception of BMI, subjects in the more recent ESTHER cohort have somewhat “healthier” lifestyles than in the other cohorts with lower smoking rates and higher rates of physical activity. Although ESTHER is the second largest cohort, it only contributes approximately 33 % of the 233,406 total person years of follow-up, because it is the most recently established cohort. The Supplementary Material (Online Resource) provides the Hazard Ratios for each major lifestyle risk factor (Tables S1–S3). These are used, as described in the detailed statistical methods (Online Resource), to derive the impact of the lifestyle related risk factors, accounting for other covariates, on LE_tot_, LE free of CVD and LE with CVD (after first event).

**Table 1:** Characteristics of the CHANCES cohorts

Baseline characteristic	Cohort		
	RCPH (Denmark)	ESTHER (Germany)	Tromsø (Norway)
Baseline year	1982–1984	2000–2002	1994–1995
Baseline total N	1759	8482	9179
Sex, males (%)	51.2	43.7	45.0
Age, mean (SD)	54.9 (5.0)	61.8 (6.6)	62.4 (9.5)
Systolic blood pressure (mmHg), mean (SD)	130.2 (18.2)	139.7 (19.5)	146.5 (23.3)
Diastolic blood pressure (mmHg), mean (SD)	80.1 (10.6)	83.8 (10.3)	84.0 (13.1)
Hypertension, yes (%)	32.3	56.4	58.9
Total cholesterol (mmol/L), mean (SD)	6.2 (1.2)	5.7 (1.3)	6.7 (1.3)
HDL-cholesterol (mmol/L), mean (SD)	1.5 (0.5)	1.4 (0.4)	1.6 (0.4)
Total/HDL cholesterol ratio, mean (SD)	4.4 (1.6)	4.5 (1.6)	4.6 (1.5)
Vigorous physical activity, yes (%)	18.1	43.8	33.6
Prevalent diabetes^a^, yes (%)	3.0	15.9	2.4
Body mass index (%)			
Underweight, <18.5	1.5	0.4	1.4
Normal, ≥18.5 and <25	47.8	27.8	41.0
Overweight, ≥25 and <30	38.0	47.1	42.8
Obese, ≥30	12.7	24.5	14.7
Alcohol^b^ (%)			
Abstainer	17.3	28.4	25.5
Light/moderate	78.9	62.6	51.3
Heavy	3.8	0.3	0.04
Smoking (%)			
Never daily smoker	39.7	49.8	34.6
Former daily smoker	16.5	31.3	33.8
Current daily smoker	42.6	16.5	31.5
Mean follow-up, range (years)	21.0, 0.1–27.2	9.1, 0.06–10.5	13.0, 0.1–16.3
Total person-years	36,931.1	77,386.1	119,089.1

Original baseline data (no imputation); excludes history of CVD and <50 years old at baseline; % do not always round to 100 % due to missing values before imputation

^a^Documented (ESTHER) or self-reported (RCPH & Tromsø)

^b^Light/moderate = men (>0 g and <60 g daily), women (>0 g and <40 g daily); Heavy = men (≥60 g daily), women (≥40 g daily)

The effects on life expectancies of each risk factor are illustrated in Tables 2, 3 and 4 for each sex separately at 50 years old with levels of the other covariates set to the mean values in the cohort. Across all three cohorts, remaining a non-smoker yielded the largest positive differences in LE_tot_, when compared to the effects of routinely taking vigorous physical activity, being overweight but not obese, and drinking in moderation. For example, among RCPH men there were: 5.8 more LE_tot_ years from remaining a non-smoker compared to 3.1 more LE_tot_ years from routinely taking vigorous physical activity, 0.9 more LE_tot_ years from being overweight but not obese, and 2.7 more LE_tot_ years from drinking in moderation compared to heavy drinkers. Among ESTHER women: 9.2 more LE_tot_ years from remaining a non-smoker compared to 6.9 more LE_tot_ years from routinely taking vigorous physical activity, 3.9 more LE_tot_ years from being overweight but not obese, and 6.0 more LE_tot_ years from drinking in moderation compared to heavy drinkers. The largest proportion of LE_tot_ in these three CHANCES cohorts is attributed to disease free years. For example, in Tromsø men: never smokers have 23.9 LE years free of CVD and 3.8 LE years *with* CVD. RCPH women who routinely take vigorous physical activity have 26.4 LE years free of CVD and 3.0 LE years with CVD.

**Table 2:** Total life expectancy (total LE), CVD-free life expectancy (LE free of CVD) and life expectancy with CVD (LE with CVD), and difference, with confidence intervals, in years at age 50, men and women, RCPH (Denmark)

	Total LE (years)	Dif total LE (years)	LE free of CVD (years)	Dif LE free of CVD (years)	LE with CVD (years)	Dif LE with CVD (years)
*Men*						
Smoking						
Never	25.3 (23.8; 26.9)	5.8 (4.3; 7.5)	22.6 (21.2; 24.2)	5.5 (4.1; 7.0)	2.7 (2.1; 3.3)	0.3 (−0.3; 1.0)
Former	25.3 (23.8; 26.7)	5.9 (4.3; 7.4)	22.6 (21.2; 24.0)	5.5 (4.1; 6.9)	2.7 (2.2; 3.3)	0.3 (−0.3; 1.0)
Current	19.4 (18.5; 20.4)	Ref	17.1 (16.2; 18.1)	Ref	2.3 (1.9; 2.9)	Ref
Physical activity (PA)						
Vigorous PA	25.0 (23.2; 26.9)	3.1 (1.4; 5.1)	22.0 (20.4; 23.9)	2.5 (0.8; 4.3)	3.0 (2.2; 4.0)	0.6 (−0.2; 1.6)
No vigorous PA	21.9 (20.9; 22.8)	Ref	19.5 (18.5; 20.4)	Ref	2.4 (2.0; 2.8)	Ref
BMI^a^						
Underweight	16.8 (13.3; 21.4)	−5.8 (−9.6; −1.3)	14.0 (11.0; 17.5)	−6.2 (−9.2; −2.5)	2.6 (0.9; 6.8)	0.1 (−1.6; 4.3)
Normal	22.6 (21.4; 23.9)	Ref	20.2 (19.0; 21.4)	Ref	2.5 (1.9; 3.1)	Ref
Overweight	23.6 (22.2; 25.0)	0.9 (−0.5; 2.4)	21.2 (19.9; 22.6)	1.1 (−0.3; 2.4)	2.4 (1.8; 3.0)	−0.1 (−0.8; 0.6)
Obese	22.3 (20.5; 24.2)	−0.3 (−2.0; 1.6)	20.2 (18.4; 22.0)	0.1 (−1.6; 2.0)	2.1 (1.5; 3.0)	−0.4 (−1.2; 0.5)
Alcohol intake^b^						
Abstainer	21.6 (19.8; 23.2)	−1.6 (−3.3; 0.0)	19.5 (17.7; 21.2)	−1.0 (−2.6; 0.6)	2.0 (1.4; 2.9)	−0.6 (−1.3; 0.2)
Light/moderate	23.1 (22.1; 24.1)	Ref	20.4 (19.4; 21.4)	Ref	2.7 (2.2; 3.2)	Ref
Heavy	20.4 (17.6; 23.1)	−2.7 (−5.3; 0.2)	18.6 (16.0; 21.5)	−1.8 (−4.4; 1.2)	1.6 (0.8; 3.2)	−1.0 (−2.0; 0.6)
*Women*						
Smoking						
Never	29.2 (27.5; 31.1)	6.4 (4.7; 8.1)	26.6 (24.9; 28.4)	6.1 (4.5; 7.8)	2.6 (2.0; 3.4)	0.3 (−0.4; 1.0)
Former	29.2 (27.4; 31.0)	6.3 (4.7; 8.2)	26.5 (24.9; 28.4)	6.0 (4.4; 7.8)	2.6 (2.0; 3.4)	0.3 (−0.4; 1.0)
Current	22.8 (21.5; 24.1)	Ref	20.5 (19.2; 21.7)	Ref	2.3 (1.8; 3.0)	Ref
Physical activity (PA)						
Vigorous PA	29.5 (27.1; 31.7)	3.5 (1.4; 5.4)	26.4 (24.3; 28.5)	2.9 (0.9; 4.8)	3.0 (2.1; 4.1)	0.6 (−0.3; 1.6)
No vigorous PA	26.0 (24.8; 27.3)	Ref	23.5 (22.4; 24.9)	Ref	2.4 (1.9; 3.0)	Ref
BMI^a^						
Underweight	19.9 (16.0; 23.9)	−6.3 (−10.4; −2.1)	17.0 (13.6; 20.4)	−6.8 (−10.5; −3.2)	2.7 (1.0; 6.2)	0.2 (−1.6; 3.6)
Normal	26.3 (24.9; 27.8)	Ref	23.8 (22.4; 25.3)	Ref	2.5 (1.9; 3.1)	Ref
Overweight	27.3 (25.6; 29.1)	1.0 (−0.7; 2.5)	24.9 (23.2; 26.6)	1.1 (−0.5; 2.7)	2.3 (1.8; 3.1)	−0.1 (−0.7; 0.5)
Obese	26.1 (23.9; 28.2)	−0.2 (−2.3; 1.9)	24.0 (21.9; 25.9)	0.2 (-1.8; 2.2)	2.1 (1.4; 3.2)	−0.4 (−1.2; 0.5)
Alcohol intake^b^						
Abstainer	25.2 (23.5; 27.0)	−1.7 (−3.5; 0.0)	23.2 (21.6; 25.0)	−1.1 (−2.8; 0.6)	2.0 (1.5; 2.8)	−0.6 (−1.4; 0.1)
Light/moderate	27.0 (25.7; 28.4)	Ref	24.3 (23.0; 25.8)	Ref	2.6 (2.1; 3.4)	Ref
Heavy	23.9 (20.9; 27.7)	−2.9 (−6.1; 0.1)	22.1 (19.2; 25.9)	−2.0 (−5.1; 1.2)	1.7 (0.8; 3.3)	−1.0 (−2.0; 0.5)

Excludes history of CVD and <50 years old at baseline

All corrected for age, history of diabetes, hypertension and total/HDL cholesterol ratio. Individually corrected for smoking status, physical activity, BMI and alcohol intake depending on model, i.e. Smoking corrected for physical activity, BMI and alcohol intake; BMI corrected for smoking status, physical activity, and alcohol intake

^a^Underweight = <18.5 kg/m^2^; normal = ≥18.5 and <25; overweight = ≥ 25 & < 30; obese = ≥ 30

^b^Light/moderate = men (>0 g and <60 g daily), women (>0 g and <40 g daily); Heavy = men (≥60 g daily), women (≥40 g daily)

**Table 3:** Total life expectancy (total LE), CVD-free life expectancy (LE free of CVD) and life expectancy with CVD (LE with CVD), and difference, with confidence intervals, in years at age 50, men and women, ESTHER (Germany)

	Total LE (years)	Dif Total LE (years)	LE free of CVD (years)	Dif LE free of CVD (years)	LE with CVD (years)	Dif LE with CVD (years)
*Men*						
Smoking						
Never	34.7 (32.5; 39.2)	8.4 (5.5; 11.5)	28.3 (27.0; 29.6)	8.9 (7.4; 10.4)	6.4 (4.3; 10.8)	−0.4 (−3.3; 2.3)
Former	31.8 (29.8; 35.8)	5.6 (2.6; 8.3)	26.1 (25.0; 27.2)	6.7 (5.3; 8.1)	5.7 (4.1; 9.7)	−1.1 (−4.0; 1.5)
Current	26.3 (23.4; 31.3)	Ref	19.4 (18.2; 20.5)	Ref	6.8 (4.3; 11.9)	Ref
Physical activity (PA)						
Vigorous PA	34.8 (31.7; 40.4)	6.8 (4.3; 10.7)	27.0 (25.8; 28.3)	4.3 (3.0; 5.7)	7.8 (4.9; 13.4)	2.4 (0.2; 6.3)
No vigorous PA	28.1 (26.3; 31.2)	Ref	22.7 (21.7; 23.6)	Ref	5.4 (3.8; 8.3)	Ref
BMI^a^						
Underweight	22.7 (16.1; 36.0)	−7.8 (−14.9; 5.2)	17.9 (13.5; 22.7)	−7.2 (−11.6; −2.0)	4.2 (0.3; 18.7)	−1.4 (−6.4; 12.9)
Normal	30.3 (28.0; 34.0)	Ref	24.9 (23.7; 26.3)	Ref	5.3 (3.3; 9.2)	Ref
Overweight	34.2 (31.3; 39.5)	3.9 (1.3; 7.3)	26.6 (25.5; 27.7)	1.7 (0.1; 3.0)	7.6 (4.8; 12.8)	2.2 (−0.2; 5.7)
Obese	31.4 (28.6; 36.4)	1.0 (−1.6; 4.3)	25.4 (23.9; 26.9)	0.4 (−1.3; 2.1)	5.9 (3.5; 11.2)	0.5 (−1.7; 3.8)
Alcohol intake^b^						
Abstainer	29.7 (27.2; 33.9)	−3.1 (−5.5; −0.9)	23.5 (22.0; 24.7)	−2.8 (−4.1; −1.5)	6.2 (3.9; 10.4)	−0.4 (−2.7; 1.8)
Light/moderate	32.8 (30.5; 37.4)	Ref	26.2 (25.3; 27.1)	Ref	6.6 (4.5; 10.8)	Ref
Heavy	27.3 (17.7; 45.2)	−5.6 (−15.5; 12.0)	21.7 (15.4; 29.0)	−4.4 (−11.1; 2.8)	4.3 (0.0; 25.2)	−2.3 (−9.1; 17.9)
*Women*						
Smoking						
Never	40.1 (37.3; 44.4)	9.2 (6.0; 11.5)	34.1 (32.7; 35.7)	9.7 (8.2; 11.5)	6.0 (3.7; 9.9)	−0.6 (−3.3; 1.6)
Former	37.0 (34.2; 41.2)	5.9 (2.9; 8.5)	31.5 (30.0; 33.3)	7.2 (5.7; 8.8)	5.3 (3.4; 8.8)	−1.2 (−4.1; 0.9)
Current	30.9 (27.8; 36.5)	Ref	24.4 (22.9; 25.9)	Ref	6.6 (3.7; 11.8)	Ref
Physical activity (PA)						
Vigorous PA	42.3 (38.6; 47.4)	6.9 (4.5; 9.9)	34.8 (33.1; 36.6)	4.8 (3.3; 6.4)	7.4 (4.3; 12.9)	2.0 (0.1; 5.2)
No vigorous PA	35.3 (33.1; 39.0)	Ref	29.9 (28.9; 31.1)	Ref	5.4 (3.5; 8.9)	Ref
BMI^a^						
Underweight	29.1 (22.4; 40.4)	−8.5 (−15.8; 3.2)	24.4 (19.1; 30.0)	−7.8 (−13.3; −2.1)	4.0 (0.3; 17.3)	−1.2 (−6.6; 12.8)
Normal	37.5 (34.6; 40.9)	Ref	32.2 (30.6; 33.8)	Ref	5.2 (3.0; 8.6)	Ref
Overweight	41.5 (37.8; 45.8)	3.9 (1.3; 6.5)	34.1 (32.4; 36.0)	1.9 (0.3; 3.6)	7.3 (4.1; 11.6)	1.9 (−0.1; 4.5)
Obese	38.7 (34.6; 43.4)	1.2 (−1.5; 4.2)	32.7 (31.0; 34.7)	0.6 (−1.2; 2.3)	5.9 (3.1; 10.2)	0.6 (−1.5; 3.3)
Alcohol intake^b^						
Abstainer	36.0 (33.3; 40.0)	−3.2 (−5.5; −1.0)	30.1 (28.7; 31.4)	−3.0 (−4.6; −1.7)	6.0 (3.7; 10.0)	−0.2 (−2.1; 1.6)
Light/moderate	39.4 (36.6; 43.4)	Ref	33.1 (31.7; 34.6)	Ref	6.3 (3.8; 9.9)	Ref
Heavy	33.3 (23.7; 48.0)	−6.0 (−16.4; 8.5)	27.9 (20.7; 35.4)	−5.1 (−12.8; 2.4)	4.2 (0.0; 21.6)	−2.0 (−8.3; 14.8)

Excludes history of CVD and <50 years old at baseline

All corrected for age, history of diabetes, hypertension and total/HDL cholesterol ratio. Individually corrected for smoking status, physical activity, BMI and alcohol intake depending on model, i.e. Smoking corrected for physical activity, BMI and alcohol intake; BMI corrected for smoking status, physical activity, and alcohol intake

^a^Underweight = <18.5 kg/m^2^; normal = ≥18.5 and <25; overweight = ≥ 25 and <30; obese = ≥30

^b^Light/moderate = men (>0 g and <60 g daily), women (>0 g and <40 g daily); Heavy = men (≥60 g daily), women (≥40 g daily)

**Table 4:** Total life expectancy (total LE), CVD−free life expectancy (LE free of CVD) and life expectancy with CVD (LE with CVD), and difference, with confidence intervals, in years at age 50, men and women, Tromsø (Norway)

	Total LE (years)	Dif total LE (years)	LE free of CVD (years)	Dif LE free of CVD (years)	LE with CVD (years)	Dif LE with CVD (years)
*Men*						
Smoking						
Never	27.7 (27.0; 28.4)	4.2 (3.4; 5.0)	23.9 (23.3; 24.6)	4.9 (4.2; 5.6)	3.8 (3.4; 4.2)	−0.7 (−1.3; −0.2)
Former	27.3 (26.7; 27.9)	3.8 (3.0; 4.5)	23.6 (23.0; 24.1)	4.5 (3.8; 5.2)	3.7 (3.3; 4.1)	−0.8 (−1.3; −0.3)
Current	23.5 (22.9; 24.2)	Ref	19.0 (18.5; 19.6)	Ref	4.5 (4.0; 5.0)	Ref
Physical activity (PA)						
Vigorous PA	27.8 (27.1; 28.4)	3.2 (2.4; 3.9)	23.0 (22.4; 23.7)	2.5 (1.7; 3.2)	4.7 (4.2; 5.3)	0.7 (0.2; 1.4)
No vigorous PA	24.6 (24.0; 25.1)	Ref	20.6 (20.0; 21.1)	Ref	4.0 (3.6; 4.4)	Ref
BMI^a^						
Underweight	21.5 (19.7; 23.2)	−4.2 (−5.9; −2.3)	19.2 (17.3; 21.0)	−3.0 (−5.0; −1.3)	2.2 (1.3; 3.8)	−1.1 (−2.2; 0.4)
Normal	25.6 (25.0; 26.2)	Ref	22.3 (21.7; 22.9)	Ref	3.3 (3.0; 3.7)	Ref
Overweight	27.0 (26.4; 27.6)	1.4 (0.7; 2.0)	22.5 (21.9; 23.1)	0.2 (−0.5; 0.9)	4.5 (4.0; 5.0)	1.1 (0.6; 1.7)
Obese	26.4 (25.5; 27.4)	0.9 (−0.1; 1.7)	21.7 (20.8; 22.6)	−0.6 (−1.5; 0.3)	4.8 (4.1; 5.5)	1.4 (0.8; 2.2)
Alcohol intake^b^						
Abstainer	26.0 (25.3; 26.7)	−0.4 (−1.1; 0.3)	21.6 (20.9; 22.3)	−0.7 (−−1.3; 0.0)	4.4 (3.9; 5.0)	0.3 (−0.3; 0.9)
Light/moderate	26.4 (25.9; 26.8)	Ref	22.2 (21.8; 22.7)	Ref	4.2 (3.8; 4.6)	Ref
Heavy	25.1 (13.0; 35.7)	−1.4 (−13.4; 9.3)	19.5 (8.5; 34.4)	−2.7 (−13.7; 12.0)	4.0 (0.5; 14.2)	−0.3 (−3.7; 10.0)
*Women*						
Smoking						
Never	31.3 (30.8; 32.0)	4.3 (3.4; 5.1)	28.6 (28.1; 29.3)	5.0 (4.2; 5.7)	2.7 (2.4; 3.0)	−0.7 (−1.1; −0.2)
Former	30.8 (30.1; 31.5)	3.8 (3.0; 4.6)	28.2 (27.5; 28.8)	4.5 (3.7; 5.3)	2.7 (2.4; 3.0)	−0.7 (−1.1; −0.3)
Current	27.0 (26.3; 27.7)	Ref	23.7 (23.0; 24.3)	Ref	3.3 (2.9; 3.8)	Ref
Physical activity (PA)						
Vigorous PA	32.6 (31.7; 33.4)	3.5 (2.7; 4.3)	28.9 (28.1; 29.7)	2.9 (2.1; 3.8)	3.6 (3.2; 4.2)	0.6 (0.1; 1.0)
No vigorous PA	29.1 (28.6; 29.5)	Ref	26.0 (25.5; 26.5)	Ref	3.1 (2.8; 3.4)	Ref
BMI^a^						
Underweight	25.1 (23.2; 26.9)	−4.4 (−6.4; −2.5)	23.4 (21.4; 25.3)	−3.6 (−5.8; −1.7)	1.6 (0.9; 2.9)	−0.8 (−1.6; 0.4)
Normal	29.5 (29.0; 30.1)	Ref	27.0 (26.5; 27.7)	Ref	2.5 (2.2; 2.8)	Ref
Overweight	31.0 (30.2; 31.7)	1.5 (0.7; 2.2)	27.6 (26.9; 28.3)	0.5 (−0.2; 1.3)	3.4 (3.0; 3.8)	0.9 (0.5; 1.3)
Obese	30.3 (29.4; 31.2)	0.8 (−0.1; 1.7)	26.7 (25.8; 27.6)	−0.3 (−1.3; 0.6)	3.6 (3.1; 4.2)	1.2 (0.7; 1.7)
Alcohol intake^b^						
Abstainer	29.7 (29.0; 30.4)	−0.5 (−1.2; 0.2)	26.4 (25.8; 27.1)	−0.7 (−1.4; 0.0)	3.3 (2.8; 3.7)	0.2 (−0.2; 0.7)
Light/moderate	30.2 (29.7; 30.7)	Ref	27.1 (26.6; 27.7)	Ref	3.0 (2.7; 3.4)	Ref
Heavy	29.2 (15.3; 41.8)	−0.9 (−15.0; 11.6)	25.0 (12.1; 40.2)	−2.1 (−15.0; 13.0)	2.8 (0.3; 11.8)	−0.2 (−2.7; 8.9)

Excludes history of CVD and <50 years old at baseline

All corrected for age, history of diabetes, hypertension and total/HDL cholesterol ratio. Individually corrected for smoking status, physical activity, BMI and alcohol intake depending on model, i.e. Smoking corrected for physical activity, BMI and alcohol intake; BMI corrected for smoking status, physical activity, and alcohol intake

^a^Underweight = <18.5 kg/m^2^; normal = ≥18.5 and <25; overweight = ≥25 and <30; obese = ≥30

^b^Light/moderate = men (>0 g and <60 g daily), women (>0 g and <40 g daily); Heavy = men (≥60 g daily), women (≥40 g daily)

Compared to the other cohorts, ESTHER had the largest positive differences in LE from participating in vigorous physical activity, even among those active after a first event. For example, ESTHER men: 6.8 more LE_tot_ years and 4.3 more LE years free of CVD, and 2.4 more LE years with CVD; RCPH men: 3.1 more LE_tot_ years; 2.5 more LE years free of CVD, and 0.6 more LE years with CVD.

Each of the cohorts displayed a survival advantage, in terms of LE_tot_ among those in the overweight category. For example, ESTHER men and women respectively; 3.9 more LE_tot_ years from being overweight and 1.0 and 1.2 more LE_tot_ years from being obese. Also, the obese in the Tromsø cohort had an apparent longevity advantage after an incident CVD event (Table 4); in men and women respectively, the obese had 4.8 and 3.6 LE years compared to 4.5 and 3.4 LE years with CVD for the overweight participants. More than 1 year lived free of CVD in the overweight, compared to those with normal BMI (both sexes) was observed in all cohorts except Tromsø.

RCPH and ESTHER exhibited a survival advantage from light/moderate alcohol intake, though the absolute magnitude of the LE_tot_ benefit varies between ~3 and ~6 years when comparing heavy with light/moderate drinkers and between ~1 and ~3 years when light/moderate drinkers are compared to abstainers. A similar survival advantage was observed in Tromsø, but was relatively negligible when comparing light/moderate drinkers and abstainers (~0.5 for both sexes), and still evident when comparing light/moderate drinkers and heavy drinkers (1.4 and 0.9 more LE_tot_ years for men and women, respectively).

The similarities and contrasts across cohorts are illustrated in Figures S1a-f (Online Resource).

A joint analysis grouped men and women into two lifestyle categories: “favourable” versus “unfavourable” and the results are shown in Table 5. The difference in LE_tot_ between these two groups ranges from ~7 years among men in Tromsø to ~16 years among women in the ESTHER study. While most of the differences in LE are in terms of life-years free of CVD, those with favourable lifestyles after a CVD event tended to live between 1 and 2 years longer in the Danish and German cohorts, which was not seen in the Norwegian cohort.

**Table 5:** Total life expectancy (total LE), CVD-free life expectancy (LE free of CVD) and life expectancy with CVD (LE with CVD), and difference, with confidence intervals, in years at age 50, men and women

	Number who fit characteristics at baseline	Total LE (years)	Dif Total LE (years)	LE free of CVD	Dif LE free of CVD (years)	LE with CVD (years)	Dif LE with CVD (years)
*RCPH*							
Men							
Favourable^a^	34	28.8 (26.3; 31.3)	9.2 (6.8; 11.6)	25.5 (23.2; 28.1)	8.3 (6.1; 10.6)	3.2 (2.3; 4.5)	0.8 (0.0; 3.4)
Unfavourable^b^	121	19.6 (18.4; 20.9)	Ref	17.2 (16.1; 18.5)	Ref	2.4 (1.8; 3.0)	Ref
Women							
Favourable	9	33.2 (30.7; 36.2)	9.7 (7.4; 12.6)	29.7 (27.0; 32.5)	8.8 (6.6; 11.5)	3.5 (2.3; 5.3)	0.9 (−0.2; 4.0)
Unfavourable	60	23.5 (21.7; 25.3)	Ref	20.8 (19.3; 22.6)	Ref	2.6 (1.9; 3.5)	Ref
*ESTHER*							
Men							
Favourable	333	40.9 (36.8; 46.8)	15.1 (11.0; 20.4)	31.6 (29.9; 33.5)	13.0 (11.1; 15.2)	9.2 (5.2; 15.2)	2.2 (−0.3; 7.3)
Unfavourable	124	25.5 (22.4; 31.1)	Ref	18.6 (17.3; 19.9)	Ref	7.0 (4.1; 12.3)	Ref
Women							
Favourable	300	47.1 (42.3; 52.5)	15.7 (11.7; 19.8)	38.5 (36.3; 41.0)	14.4 (12.2; 16.8)	8.4 (4.4; 14.1)	1.5 (−0.6; 5.8)
Unfavourable	81	31.1 (27.3; 37.1)	Ref	24.1 (22.3; 25.9)	Ref	6.9 (3.7; 13.0)	Ref
*Tromsø*							
Men							
Favourable	167	30.4 (29.5; 31.4)	7.4 (6.3; 8.4)	25.6 (24.8; 26.6)	7.4 (6.4; 8.5)	4.8 (4.2; 5.4)	0.1 (−0.4; 0.6)
Unfavourable	288	23.0 (22.3; 23.8)	Ref	18.2 (17.5; 18.9)	Ref	4.8 (4.2; 5.6)	Ref
Women							
Favourable	155	34.9 (33.9; 35.9)	7.8 (6.8; 9.0)	31.1 (30.1; 32.1)	8.1 (6.9; 9.2)	3.8 (3.3; 4.5)	0.0 (−0.4; 0.4)
Unfavourable	255	27.1 (26.3; 27.9)	Ref	23.0 (22.2; 23.8)	Ref	4.0 (3.5; 4.7)	Ref

Excludes history of CVD and <50 years old at baseline

When using the repeated measures of covariates in the Markov model, the most recent available value for each measurement was used in the analysis

^a^BMI overweight (not obese), light/moderate drinker, non-smoker, and partakes in vigorous physical activity

^b^BMI overweight (not obese), light/moderate drinker, smoker, and does not partakes in vigorous physical activity

## Discussion

The reduction in mortality rates from CVD, over more than three decades in some western European countries, is a public health success story. Though the contribution to this decline by changes in CVD *incidence* secondary to changes in risk factor prevalence is somewhat contested, consensus has emerged that the majority of the decline has been due to changes in lifestyle related risk factors, rather than treatment [22, 23].

Our findings bear similarities to some previous studies [6], but by accounting for repeated measures of lifestyle factors within our multi-state transition model, we have methodologically extended previous work. Analyses that use only a single baseline assessment of lifestyle cannot account for possible changes in these, which may lead to a biased estimation of risk. These previous studies [6–9] have focused on subjects recruited at younger ages than in this analysis of CHANCES cohorts and it is important for policy makers and the public to know that prevention is still possible later in life.

Overall, some consistent patterns are discernible in the impact of lifestyle risk factors on LE_tot_ and LE free of CVD across the three cohorts that were studied. For 50 year olds, across all three cohorts, remaining a non-smoker yielded the largest positive differences in LE_tot_, when compared to the effects of routinely taking vigorous physical activity, being overweight but not obese, and drinking in moderation. This is consistent with other findings [6, 24, 25], but it should be noted that by far the largest proportion of the LE in these three CHANCES cohorts was attributed to *disease free years* emphasising the much greater population dividend from maintaining a favourable lifestyle.

It is at first surprising that at least in the ESTHER and Tromsø cohorts, smokers had an apparent (though small) longevity advantage after an incident CVD event. It should be noted that the confidence intervals are wide and this apparent difference may be spurious. It is claimed that such seemingly perverse findings may represent a form of survivorship bias [26] whereby death has harvested the “weakest” smokers who succumb to a first event, leaving those who survived as an unrepresentative but “hardy” subsample. Another interesting finding from this study was the apparent large differences in LE_tot_ between the cohorts, with ESTHER having the highest overall LE at age 50 years for both males and females, regardless of risk factor. The reason for this may be that the ESTHER cohort have somewhat “healthier” lifestyles than the other cohorts as outlined previously. Alternatively, the direction of the differences are consistent with the fact that RCPH baseline was 20 years earlier and Tromsø was 10 years earlier than ESTHER; there has been substantial increases in life expectancy over time due to medical advances and lifestyle changes, such as a sharp decline in the prevalence of smoking in recent decades.

While the majority of the life years from participating in vigorous physical activity arise in years of life lived free of CVD, there is still a material advantage observed among those active after a first event, which is consistent with the benefits reported for various cardiac rehabilitation programmes that emphasize graded physical activity [27]. There are substantial differences in the absolute magnitude of the overall survival advantage from vigorous physical activity across cohorts and a possible interpretation is likely to lie in the nature and methods of sample recruitment and measurement of physical activity, where it is apparent that far more people in ESTHER (than in for example RCPH) state that they regularly participated in regular vigorous physical activity. Of course in order to properly quantify the health benefits of regular physical activity we would require accelerometry, which was not available when, for example, the RCPH study commenced.

Each of our cohorts showed a survival advantage, in terms of LE_tot_, among those in the overweight category. More than 1 year lived free of CVD in the overweight, compared to those with normal BMI (both sexes) was observed in all cohorts except Tromsø. The smaller number of obese subjects and wider confidence intervals signifies that their overall LE is difficult to distinguish from those of normal weight in our cohorts, although the underweight subjects fare significantly worse and this can commonly be explained by subclinical or occult diseases, smoking, sarcopenia, and frailty [28–30]. As it is difficult to fully account for all such conditions, further research on this group of people is warranted, particularly in the older population. The years of life lived after a CVD event are likewise higher in the overweight subjects than among the normal weight subjects at least in the ESTHER and Tromsø cohorts, by around one and 2 years, respectively, though this trend is not apparent in the RCPH data. This greater survival after an event among the overweight has been observed by others [9]. Paradoxically, the obese in Tromsø displayed more years lived after a CVD event, greater than 1 year for both sexes, compared to the normal and overweight categories. This could potentially reflect the hypothesised “obesity paradox”, but the existence of such has been disputed [31]. We cannot establish the extent to which the association between obesity and number of years lived *free of* and *with* CVD is causal. Several hypotheses have been put forward to explain such findings. Heavier individuals may present earlier for medical treatment for obesity related conditions including cholesterol reduction, diabetes and hypertension [32]. Alternatively, there may be “cardioprotective” metabolic effects of increased body fat in times of chronic illness [33]. Small increases in BMI (e.g. normal → overweight category) can be due to an increased lean mass which may be associated with improved metabolic profiles and better prognosis in relation to chronic illness and mortality [32].

All the cohorts’ accord well in pointing to a survival advantage from light/moderate alcohol intake, though the absolute magnitude of the LE_tot_ benefit varies as outlined in the results. A majority of the benefit from moderate intake appears to arise from a reduced incidence of events and a greater event free survival, but there still appears to be a survival benefit after disease onset. While the reported J-shaped curve between intake and CVD mortality has been thought to arise in part from subsamples of non-drinkers who gave up because of some ill health effects, this would not be a powerful explanation for our own findings, since we accommodated repeated measures of behaviours in our analysis. On the other hand, we acknowledge that we have only crudely categorised “heavy” drinkers and have not been able, with this data, to tease out any distinct effects of binge drinking [34]. Nor did our sample size justify a greater number of Markov states to separate coronary heart disease events from stroke events, and the effects of heavy drinking are likely to be stronger on stroke incidence and mortality [35, 36].

All our cohorts demonstrate the sizeable benefits to LE *without* CVD and also for survival after CVD onset, when people favour a lifestyle characterized by salutary behaviours: not smoking, light/moderate drinking, taking regular exercise, and a modest excess BMI. Those who have a favourable lifestyle live between ~8 and ~16 years longer overall, of which (with the exception of Tromsø) between ~1 and ~2 extra years is apparent after an event. These values accord broadly with those reported by Nusselder et al. [6], but the population in the latter Framingham study was younger at baseline (28–62 years) and so an important message from our results is that the LE benefits of maintaining a favourable lifestyle applies among older subjects as well as the young. Some differences in these estimates from those in other studies might be expected, as we have derived them by setting other covariates to their mean value and the distribution of such variables will vary from population to population.

Some limitations of our study need to be considered. Although all data were harmonised based on agreed rules (www.chancesfp7.eu; [14, 18]), the data from the different cohorts are not perfectly comparable, due to differences in study design and data collection procedures, with the potential for residual inconsistencies in variable definitions, e.g. retrospective standardization of physical activity data is known to be very difficult, and there have been major difficulties in standardizing physical activity questionnaires across countries [18]. Additionally, not all detailed endpoints, including all CVDs were possible to be coded within all CHANCES cohorts due to data availability, including non-CVDs. Therefore, we could not completely take into account all other competing risks within the current analysis. Because of our desire to incorporate repeated measures of risk factors, which previous similar studies have not yet attempted, we did not consider it useful or feasible to conduct an individual subject meta-analysis, as the intervals between follow-up examinations in the cohorts were different. When using the repeated measures of covariates in the Markov model, the most recent available value for each measurement was used in the analysis. However, the repeated measures were not always taken frequently and in many cases of non-fatal CVD (state 2) the last measurement was taken when the person was in state 1 (recontact dependent with some participants having measurements taken closer to the event than others). Such measurements may not wholly reflect the risk factor levels at state 2 because after a nonfatal CVD event, a person is going to be under aggressive intervention to alter his/her risk factors, and that person is usually motivated to change their lifestyle. Nevertheless, this model is much better and more robust than simply relying solely on the baseline measurements of the risk factors. For example, smoking is known to advance death, so having repeated measures we can take the most recent available measurement so as to account for someone who may become an ex-smoker after moving to state 2 rather than assuming they remain a smoker if we just used baseline measurements. Furthermore, CHANCES has no data on the acute treatment of the incident events. While it is accepted that salutary behaviours have benefits of comparable magnitude to many treatments [37], treatment effects in this phase of disease may clearly confound the effects of the lifestyle risk factors. While Ko et al. propose [38], and demonstrate empirically, that older patients with shorter LE actually receive evidence based treatments less frequently than younger subjects, we have no basis for thinking that acute CVD treatments after an event are correlated with our examined risk factors in these cohorts. Insofar as some treatments might plausibly be offered more frequently to higher risk patients than lower risk patients (e.g. obese vs. lean patients receiving more careful monitoring or treatment with blood pressure lowering agents), the benefits of lifestyle change might be over-estimated, though the final direction and significance of confounding by treatment effects (after disease onset) in our study is unknown. Studies have consistently demonstrated that abdominal obesity may be a better predictor for mortality and disease outcomes than overall obesity [39, 40]. This may be particularly relevant in the elderly due to age-related changes in body composition, such as a decrease in muscle mass, increase in fat mass, and loss of height [41]. Regardless, BMI continues to be widely used in epidemiological studies and it was universally available across all cohorts and follow-ups unlike other measures of adiposity such as waist-to-hip ratio. Although sex-differences in our analyses were not always apparent, we chose to present sex-stratified results due to differences in the lifestyle risk factors among the individual cohorts, and also due to a priori understanding that the impact of various risk factors, such as smoking is different between men and women [42].

In conclusion, there are sizeable benefits to LE without CVD and also for survival after CVD onset when people favour a lifestyle characterized by salutary behaviours. Remaining a non-smoker yielded the greatest extra years in overall LE, when compared to the effects of routinely taking physical activity, being overweight but not obese, and drinking in moderation. The majority of the overall LE benefit is in disease free years. Given the higher incidence of cardiovascular events and mortality in older age, lifestyle choices in the older population could probably achieve even greater absolute risk reductions for adverse cardiovascular events. Perceptions of LE are associated with a variety of health-related behaviours [43] and so it is important that the benefits of maintaining a favourable lifestyle are known by older subjects. Additionally, having a means of showing LE with and without disease may be a useful communication tool for this section of the population [44].

## Electronic supplementary material

Below is the link to the electronic supplementary material.

Supplementary material 1 Contains detailed statistical methods, along with supplementary tables illustrating the hazard ratios for the different transitions for each cohort and figures of the effects on life expectancies of each risk factor for each sex separately at 50 years old. (PDF 877 kb)

## References

[1] World Health Organization. Fact sheet No. 317: Cardiovascular diseases (CVDs). Available from World Heal. Organ. site, http//www.who.int/mediacentre/factsheets/fs317/en/. 2013.

[2] Mathers CD, Loncar D (2006). Projections of global mortality and burden of disease from 2002 to 2030. PLoS Med.

[3] Jagger C, Gillies C, Moscone F, Cambois E, Van Oyen H, Nusselder W (2008). Inequalities in healthy life years in the 25 countries of the European Union in 2005: a cross-national meta-regression analysis. Lancet.

[4] Fries JF (1980). Aging, natural death, and the compression of morbidity. Bull World Health Organ.

[5] Walter S, Kunst A, Mackenbach J, Hofman A, Tiemeier H (2009). Mortality and disability: the effect of overweight and obesity. Int J Obes (Lond)..

[6] Nusselder WJ, Franco OH, Peeters A, Mackenbach JP (2009). Living healthier for longer: comparative effects of three heart-healthy behaviors on life expectancy with and without cardiovascular disease. BMC Public Health.

[7] Al Mamun A, Peeters A, Barendregt J, Willekens F, Nusselder W, Bonneux L (2004). Smoking decreases the duration of life lived with and without cardiovascular disease: a life course analysis of the Framingham Heart Study. Eur Heart J.

[8] Franco OH, de Laet C, Peeters A, Jonker J, Mackenbach J, Nusselder W (2005). Effects of physical activity on life expectancy with cardiovascular disease. Arch Intern Med.

[9] Pardo Silva MC (2006). De Laet C, Nusselder WJ, Mamun AA, Peeters A. Adult obesity and number of years lived with and without cardiovascular disease. Obesity (Silver Spring).

[10] Ronksley PE, Brien SE, Turner BJ, Mukamal KJ, Ghali WA (2011). Association of alcohol consumption with selected cardiovascular disease outcomes: a systematic review and meta-analysis. BMJ.

[11] Costanzo S, Di Castelnuovo A, Donati MB, Iacoviello L, de Gaetano G (2010). Alcohol consumption and mortality in patients with cardiovascular disease. J Am Coll Cardiol.

[12] Di Castelnuovo A, Costanzo S, Bagnardi V, Donati MB, Iacoviello L, de Gaetano G (2006). Alcohol dosing and total mortality in men and women: an updated meta-analysis of 34 prospective studies. Arch Intern Med.

[13] Abdullah A, Wolfe R, Mannan H, Stoelwinder JU, Stevenson C, Peeters A (2012). Epidemiologic merit of obese-years, the combination of degree and duration of obesity. Am J Epidemiol.

[14] Boffetta P, Bobak M, Borsch-Supan A, Brenner H, Eriksson S, Grodstein F (2014). The Consortium on Health and Ageing: network of Cohorts in Europe and the United States (CHANCES) project-design, population and data harmonization of a large-scale, international study. Eur J Epidemiol.

[15] Osler M, Linneberg A, Glümer C, Jørgensen T (2011). The cohorts at the Research Centre for Prevention and Health, formerly “The Glostrup Population Studies”. Int J Epidemiol.

[16] Schöttker B, Müller H, Rothenbacher D, Brenner H (2013). Fasting plasma glucose and HbA1c in cardiovascular risk prediction: a sex-specific comparison in individuals without diabetes mellitus. Diabetologia.

[17] Jacobsen BK, Eggen AE, Mathiesen EB, Wilsgaard T, Njølstad I (2012). Cohort profile: the Tromso Study. Int J Epidemiol.

[18] Kuulasmaa K, Palosaari T editors. C from P of the C on H and AN of C in E and the US (CHANCES). CHANCES cohort descriptions, assessment of the availability and quality of data, and definitions of variables [Internet]. MORGAM Proj. e-publications. [cited 2015 Apr 29]. Available from: http://www.thl.fi/publications/morgam/chances_d9/index.html.

[19] Cox D, Miller H (1965). The theory of stochastic processes.

[20] Jackson CH (2011). Multi-state models for panel data: the msm Package for R. J Stat Softw.

[21] van den Hout A, Jagger C, Matthews FE (2009). Estimating Life Expectancy in Health and Ill Health by Using a Hidden Markov Model. J R Stat Soc Ser C ((Applied Stat. Wiley for the Royal Statistical Society).

[22] O’Flaherty M, Buchan I, Capewell S (2013). Contributions of treatment and lifestyle to declining CVD mortality: why have CVD mortality rates declined so much since the 1960s?. Heart.

[23] Ezzati M, Obermeyer Z, Tzoulaki I, Mayosi BM, Elliott P, Leon DA (2015). Contributions of risk factors and medical care to cardiovascular mortality trends. Nat Rev Cardiol.

[24] Goldman DP, Zheng Y, Girosi F, Michaud P-C, Olshansky SJ, Cutler D (2009). The benefits of risk factor prevention in Americans aged 51 years and older. Am J Public Health.

[25] Clarke R, Emberson J, Fletcher A, Breeze E, Marmot M, Shipley MJ (2009). Life expectancy in relation to cardiovascular risk factors: 38 year follow-up of 19,000 men in the Whitehall study. BMJ.

[26] Riggs JE (1993). Smoking and Alzheimer’s disease: protective effect or differential survival bias?. Lancet.

[27] Alter DA, Oh PI, Chong A (2009). Relationship between cardiac rehabilitation and survival after acute cardiac hospitalization within a universal health care system. Eur J Cardiovasc Prev Rehabil.

[28] Borrell LN, Samuel L (2014). Body mass index categories and mortality risk in US adults: the effect of overweight and obesity on advancing death. Am J Public Health.

[29] de Hollander EL, Bemelmans WJ, Boshuizen HC, Friedrich N, Wallaschofski H, Guallar-Castillón P (2012). The association between waist circumference and risk of mortality considering body mass index in 65- to 74-year-olds: a meta-analysis of 29 cohorts involving more than 58 000 elderly persons. Int J Epidemiol.

[30] Hubbard RE, Lang IA, Llewellyn DJ, Rockwood K (2010). Frailty, body mass index, and abdominal obesity in older people. J Gerontol A Biol Sci Med Sci.

[31] Dorner TE, Rieder A (2012). Obesity paradox in elderly patients with cardiovascular diseases. Int J Cardiol.

[32] Romero-Corral A, Montori VM, Somers VK, Korinek J, Thomas RJ, Allison TG (2006). Association of bodyweight with total mortality and with cardiovascular events in coronary artery disease: a systematic review of cohort studies. Lancet.

[33] Auyeung TW, Lee JSW, Leung J, Kwok T, Leung PC, Woo J (2010). Survival in older men may benefit from being slightly overweight and centrally obese—a 5-year follow-up study in 4,000 older adults using DXA. J Gerontol A Biol Sci Med Sci.

[34] Ruidavets J-B, Ducimetière P, Evans A, Montaye M, Haas B, Bingham A (2010). Patterns of alcohol consumption and ischaemic heart disease in culturally divergent countries: the Prospective Epidemiological Study of Myocardial Infarction (PRIME). BMJ.

[35] Hart CL, Smith GD, Hole DJ, Hawthorne VM (1999). Alcohol consumption and mortality from all causes, coronary heart disease, and stroke: results from a prospective cohort study of scottish men with 21 years of follow up. BMJ.

[36] Glynn RJ, Rosner B (2005). Comparison of risk factors for the competing risks of coronary heart disease, stroke, and venous thromboembolism. Am J Epidemiol.

[37] Iestra JA, Kromhout D, van der Schouw YT, Grobbee DE, Boshuizen HC, van Staveren WA (2005). Effect size estimates of lifestyle and dietary changes on all-cause mortality in coronary artery disease patients: a systematic review. Circulation.

[38] Ko DT, Austin PC, Tu JV, Lee DS, Yun L, Alter DA (2014). Relationship between care gaps and projected life expectancy after acute myocardial infarction. Circ CardiovascQual Outcomes.

[39] Petursson H, Sigurdsson JA, Bengtsson C, Nilsen TIL, Getz L (2011). Body configuration as a predictor of mortality: comparison of five anthropometric measures in a 12 year follow-up of the Norwegian HUNT 2 study. PLoS ONE.

[40] Haslam DW, James WPT (2005). Obesity. Lancet.

[41] Zamboni M, Mazzali G, Zoico E, Harris TB, Meigs JB, Di Francesco V (2005). Health consequences of obesity in the elderly: a review of four unresolved questions. Int J Obes (Lond).

[42] Yahagi K, Davis HR, Arbustini E, Virmani R (2015). Sex differences in coronary artery disease: pathological observations. Atherosclerosis.

[43] Ziegelmann JP, Lippke S, Schwarzer R (2006). Subjective residual life expectancy in health self-regulation. J Gerontol B Psychol Sci Soc Sci.

[44] Rubrichi S, Rognoni C, Sacchi L, Parimbelli E, Napolitano C, Mazzanti A (2015). Graphical representation of life paths to better convey results of decision models to patients. Med Decis Making.

