# Supplementary material for: Effect of major lifestyle risk factors, independent and jointly, on life expectancy with and without cardiovascular disease: results from the Consortium on Health and Ageing Network of Cohorts in Europe and the United States (CHANCES)
Source: Eur J Epidemiol. 2016 Jan 18;31:455–68. doi: 10.1007/s10654-015-0112-8 (PMC4901087; doi:10.1007/s10654-015-0112-8)
Supplement: Supplementary file 1 — Supplementary material 1 Contains detailed statistical methods, along with supplementary tables illustrating the hazard ratios for the different transitions for each cohort and figures of the effects on life expectancies of each risk factor for each sex separately at 50 years old. (PDF 877 kb) [file 10654_2015_112_MOESM1_ESM.pdf]

Title:

Effect of major lifestyle risk factors, independent and jointly, on life expectancy with and without cardiovascular disease: results from the Consortium on Health and Ageing Network of Cohorts in Europe and the United States (CHANCES).

Journal: The European Journal of Epidemiology

Authors:
O'Doherty MG (1), Cairns K (2), O'Neill V (1), Lamrock F (1, 2), Jørgensen T (3, 4, 5), Brenner H (6), Schöttker B (6), Wilsgaard T (7), Siganos G (7), Kuulasmaa K (8), Boffetta P (9, 10), Trichopoulou A (10, 11) & Kee F (1), on behalf of the CHANCES consortium.

[1]UKCRC Centre of Excellence for Public Health for Northern Ireland, Queens University Belfast, Belfast, Northern Ireland, BT12 6BA, UK.
[2]Centre for Statistical Science and Operational Research (CenSSOR), Queen's University Belfast, Belfast, Northern Ireland, BT7 1NN, UK.
[3]Research Centre for Prevention and Health, Glostrup University Hospital, Denmark.
[4]University of Copenhagen, Institute of Public Health, Denmark.
[5]University of Aalborg, Faculty of Medicine, Denmark.
[6]Division of Clinical Epidemiology and Aging Research, German Cancer Research Center (DKFZ), Im Neuenheimer Feld 581, 69120 Heidelberg, Germany.
[7]University of Tromsø, Department of Community Medicine, 9037 Tromsø, Norway.
[8]Department of Health, National Institute for Health and Welfare (THL), FI-00271, Helsinki, Finland.
[9]The Tisch Cancer Institute and Institute for Translational Epidemiology, Mount Sinai School of Medicine, New York, NY 10029, USA.
[10]Hellenic Health Foundation, Kaisareias 13 & Alexandroupoleos str., GR-115 27, Athens, Greece.
[11]University of Athens, Medical School, Department of Hygiene, Epidemiology and Medical Statistics, Mikras Asias 75 st, GR-115 27, Athens, Greece.

Address for correspondence:

Dr Mark O'Doherty
UKCRC Centre of Excellence for Public Health
Queens University
Grosvenor Road
Belfast BT12 6BJ
m.odoherty@qub.ac.uk

Supplementary Material

Detailed statistical methods

The parameters of such a three state model were estimated through use of the R msm package[1]. In msm these transition rates q_{ij} (representing the instantaneous risk of moving from state *i* to state *j*) can be influenced by the characteristics of individuals (either time-dependent or constant covariates, **z**) in a proportional hazards fashion:

$$q_{ij}(\mathbf{z}) = q_{ij}^{(0)} \exp\left(\boldsymbol{\beta}_{ij}{}^{T}\mathbf{z}\right)$$

In msm we determine the unknown parameters $q_{ij}^{(0)}$ and $\boldsymbol{\beta}_{ij}$ through maximising the likelihood expression[2].

A multiple imputation (MI) procedure was implemented to account for missingness in the covariates[3]. Missing data (after exclusion criteria was applied) ranged from 0% to ~25% across the cohorts. In this case L =30 'complete' data sets were made for each cohort, imputing missing data from the distribution of the missing data given the observed. The msm package was then utilised on each of the 30 imputed data sets, giving 30 sets of parameter estimates for $q_{ij}^{(0)}$ and $\boldsymbol{\beta}_{ij}$ (estimates for $q_{ij}^{(0)}$ and $\boldsymbol{\beta}_{ij}$ are collectively labelled $\hat{\mathbf{B}}_l$ for the lth imputed data set, with associated variance-covariance matrix $\hat{\boldsymbol{\sigma}}_l{}^2$). Rubin's rules for inference were deployed to determine the multiple imputation estimate of $\mathbf{B}$, $\hat{\mathbf{B}}_{\text{MI}}$:

$$\hat{\mathbf{B}}_{\text{MI}} = \frac{1}{L}\sum_{l=1}^{L}\hat{\mathbf{B}}_l,$$

with variance-covariance matrix given by:

$$\hat{\mathbf{V}}_{\text{MI}} = \frac{1}{L}\sum_{l=1}^{L}\hat{\boldsymbol{\sigma}}_l{}^2 + \left(1+\frac{1}{L}\right)\frac{1}{(L-1)}\sum_{l=1}^{L}\left(\hat{\mathbf{B}}_l - \hat{\mathbf{B}}_{\text{MI}}\right)^2.$$

Hazard ratios (HRs), $\exp\left(\left(\boldsymbol{\beta}_{ij}\right)_k\right)$, [indicating the effect of covariate *k* on the transition between state *i* and *j*] were thus determined, together with their corresponding confidence interval, based on their corresponding estimates within $\hat{\mathbf{B}}_{\text{MI}}$ and $\hat{\mathbf{V}}_{\text{MI}}$. [For those risk factors

having a significant independent effect on flow between states, the HR confidence interval will not span the value 1.]

Finally parameter estimates obtained were used to calculate LE with and without CVD at age 50 by risk factor level, both varying one risk factor at a time and varying many at a time. LE was calculated following the techniques of the R ELECT package[4].

The expected LE in state $s \in \{1,2\}$ given initial state $r \in \{1,2\}$ and covariates $\mathbf{z}$ (including one time dependent covariate of age - with $\text{age}(t=0)=a_0$) is given by:

$$e_{rs}\{\mathbf{z},a_0\} = \int_0^\infty \Pr\left\{X_t = s \middle| X_0 = r, \mathbf{z},\ \text{age}(t=0)=a_0\right\} dt.$$

This integral is numerically approximated using:

$$e_{rs}\{\mathbf{z},a_0\} \approx \frac{1}{2} + \sum_{g=1}^{G} \Pr\left\{X_{t=gh} = s \middle| X_0 = r, \mathbf{z},\ \text{age}(t=0)=a_0\right\} \times h,$$

where h denotes a small time interval and G is sufficiently large to ensure the integrand is negligible at $t = Gh$. In order to calculate the above integrand, the transition probability matrix $\mathbf{P}(t)$ (whose $(r,s)^{\text{th}}$ entry equals $\Pr\left\{X_t = s \middle| X_0 = r, \mathbf{z},\ \text{age}(t=0)=a_0\right\}$) is evaluated at each time point in the grid. Piecewise-constant transition rates $q_{ij}(\mathbf{z})$ are assumed within each time interval, so that:

$$\begin{aligned} &\mathbf{P}(t=gh) \\ &\approx \mathbf{P}_h\left(t_1=0, t_2=h \middle| \mathbf{z}, \text{age=}a_0\right) \times \mathbf{P}_h\left(t_1=h, t_2=2h \middle| \mathbf{z}, \text{age=}a_0+h\right) \\ &\quad \times \ldots \times \mathbf{P}_h\left(t_1=(g-1)h, t_2=gh \middle| \mathbf{z}, \text{age=}a_0+(g-1)h\right) \end{aligned}$$

where the $(r,s)^{\text{th}}$ entry of $\mathbf{P}_h\left(t_1, t_2 \middle| \mathbf{z}, \text{age=}a_g\right)$ equals $\Pr\left\{X_{t_2} = s \middle| X_{t_1} = r, \mathbf{z},\ \text{age}=a_g\right\}$. For the non-recoverable illness-death model $\mathbf{P}_h$ has the following analytic expression:

$$\mathbf{P}_h\left(t_1=\alpha, t_2=\alpha+h \middle| \mathbf{z}, \text{age}=a_g\right)=\exp\left(\mathbf{Q}\left(\mathbf{z}, a_g\right)h\right)$$

$$\begin{pmatrix} e^{-(q_{12}+q_{13})h} & \frac{q_{12}}{q_{12}+q_{13}-q_{23}}\left(e^{-q_{23}h}-e^{-(q_{12}+q_{13})h}\right) & 1-e^{-(q_{12}+q_{13})h}-\frac{q_{12}}{\left(q_{12}+q_{13}-q_{23}\right)}\left(e^{-q_{23}h}-e^{-(q_{12}+q_{13})h}\right) \\ 0 & e^{-q_{23}h} & 1-e^{-q_{23}h} \\ 0 & 0 & 1 \end{pmatrix}$$

where q_{12}, q_{13} and q_{23} are constant within each time interval - depending on $\mathbf{z}$ (in particular the given age) and the $q_{ij}^{(0)}$ and $\boldsymbol{\beta}_{ij}$ parameters as given within $\hat{\mathbf{B}}_{\text{MI}}$.

Uncertainty in the LE measures was assessed using simulation. That is, LE was calculated as described above, but samples from the multivariate normal $N\left(\hat{\mathbf{B}}_{\text{MI}}, \hat{\mathbf{V}}_{\text{MI}}\right)$ were drawn to estimate the $q_{ij}^{(0)}$ and $\boldsymbol{\beta}_{ij}$ parameters in the above scheme for each simulation.

Table S1. Hazard ratios for the different transitions, RCPH (Denmark)

	No CVD to CVD	No CVD to death	CVD to death
Number of events	403	691	253
Age	1.07 (1.05; 1.09)	1.07 (1.06; 1.09)	1.07 (1.05; 1.09)
Female	0.73 (0.59; 0.90)	0.67 (0.56; 0.79)	0.73 (0.55; 0.97)
Prevalent diabetes[a]	1.24 (0.80; 1.95)	1.47 (1.05; 2.07)	1.75 (0.98; 3.15)
Vigorous physical activity[a]	0.93 (0.71; 1.20)	0.70 (0.56; 0.87)	0.80 (0.55; 1.16)
Total/HDL cholesterol ratio	1.19 (1.13; 1.26)	0.98 (0.92; 1.03)	1.06 (0.99; 1.14)
Hypertension[a]	1.40 (1.13; 1.73)	1.26 (1.07; 1.48)	1.01 (0.78; 1.31)
Smoking			
Never	Ref	Ref	Ref
Former	-	1.01 (0.80; 1.27)	-
Current	1.50 (1.21; 1.86)	1.94 (1.59; 2.36)	1.35 (1.04; 1.75)
BMI[b]			
Underweight	1.79 (0.90; 3.58)	2.03 (1.28; 3.21)	1.17 (0.38; 3.60)
Normal	Ref	Ref	Ref
Overweight	0.82 (0.65; 1.03)	0.94 (0.79; 1.12)	0.87 (0.65; 1.17)
Obese	0.87 (0.65; 1.17)	1.04 (0.83; 1.31)	1.05 (0.71; 1.56)
Alcohol[c]			
Abstainer	0.87 (0.66; 1.14)	1.24 (1.02; 1.50)	1.11 (0.80; 1.56)
Light/moderate	Ref	Ref	Ref
Heavy	0.93 (0.53; 1.62)	1.34 (0.95; 1.89)	1.54 (0.77; 3.08)

[a]Dichotomous (Yes/No), Reference=No

[b]Underweight = <18.5 kg/m^2; normal = ≥18.5 & <25; overweight = ≥25 & <30; obese = ≥30

[c]Light/moderate = men (>0g & <60g daily), women (>0g & <40g daily); Heavy = men (≥60g daily), women (≥40g daily)

Table S2. Hazard ratios for the different transitions, ESTHER (Germany)

	No CVD to CVD	No CVD to death	CVD to death
Number of events	523	817	50
Age	1.06 (1.04; 1.07)	1.09 (1.07; 1.10)	1.08 (1.02; 1.14)
Female	0.59 (0.49; 0.70)	0.58 (0.49; 0.67)	0.55 (0.29; 1.02)
Prevalent diabetes[a]	1.35 (1.10; 1.65)	1.42 (1.21; 1.67)	3.15 (1.78; 5.58)
Vigorous physical activity[a]	0.93 (0.78; 1.12)	0.54 (0.46; 0.63)	0.61 (0.31; 1.18)
Total/HDL cholesterol ratio	1.08 (1.02; 1.15)	1.02 (0.96; 1.08)	1.11 (0.95; 1.31)
Hypertension[a]	1.59 (1.30; 1.96)	1.45 (1.23; 1.71)	1.32 (0.53; 3.27)
Smoking			
Never	Ref	Ref	Ref
Former	-	1.40 (1.17; 1.66)	-
Current	2.02 (1.63; 2.51)	2.61 (2.16; 3.15)	1.53 (0.70; 3.34)
BMI[b]			
Underweight	1.18 (0.32; 4.35)	2.39 (1.32; 4.33)	0.96 (0.02; 59.6)
Normal	Ref	Ref	Ref
Overweight	1.13 (0.90; 1.41)	0.73 (0.61; 0.86)	0.72 (0.34; 1.50)
Obese	1.17 (0.91; 1.50)	0.86 (0.71; 1.04)	1.15 (0.53; 2.49)
Alcohol[c]			
Abstainer	1.24 (1.02; 1.50)	1.33 (1.15; 1.55)	1.33 (0.73; 2.44)
Light/moderate	Ref	Ref	Ref
Heavy	0.97 (0.25; 3.76)	1.79 (0.81; 3.96)	0.98 (0.001; 816.24)

[a]Dichotomous (Yes/No), Reference=No

[b]Underweight = <18.5 kg/m^2; normal = ≥18.5 & <25; overweight = ≥25 & <30; obese = ≥30

[c]Light/moderate = men (>0g & <60g daily), women (>0g & <40g daily); Heavy = men (≥60g daily), women (≥40g daily)

Table S3. Hazard ratios for the different transitions, Tromsø (Norway)

	No CVD to CVD	No CVD to death	CVD to death
Number of events	1713	2108	727
Age	1.06 (1.06; 1.07)	1.11 (1.10; 1.12)	1.11 (1.10; 1.12)
Female	0.57 (0.51; 0.63)	0.61 (0.55; 0.67)	0.74 (0.63; 0.87)
Prevalent diabetes[a]	2.10 (1.68; 2.63)	1.85 (1.50; 2.28)	1.88 (1.43; 2.48)
Vigorous physical activity[a]	0.94 (0.85; 1.05)	0.63 (0.56; 0.70)	0.80 (0.66; 0.96)
Total/HDL cholesterol ratio	1.15 (1.12; 1.19)	1.00 (0.97; 1.03)	1.03 (0.99; 1.08)
Hypertension[a]	1.75 (1.55; 1.97)	1.14 (1.03; 1.26)	1.27 (1.03; 1.58)
Smoking			
Never	Ref	Ref	Ref
Former	1.23 (1.23; 1.23)	1.07 (0.96; 1.20)	-
Current	1.85 (1.66; 2.06)	1.60 (1.42; 1.79)	1.52 (1.28; 1.81)
BMI[b]			
Underweight	0.88 (0.53; 1.46)	1.80 (1.41; 2.31)	1.30 (0.70; 2.43)
Normal	Ref	Ref	Ref
Overweight	1.23 (1.10; 1.38)	0.81 (0.73; 0.90)	0.85 (0.72; 1.01)
Obese	1.31 (1.14; 1.51)	0.91 (0.80; 1.03)	0.78 (0.63; 0.98)
Alcohol[c]			
Abstainer	1.06 (0.95; 1.20)	1.08 (0.98; 1.19)	0.94 (0.79; 1.11)
Light/moderate	Ref	Ref	Ref
Heavy	0.94 (0.11; 8.26)	1.10 (0.15; 7.88)	-

[a]Dichotomous (Yes/No), Reference=No

[b]Underweight = <18.5 kg/m^2; normal = ≥18.5 & <25; overweight = ≥25 & <30; obese = ≥30

[c]Light/moderate = men (>0g & <60g daily), women (>0g & <40g daily); Heavy = men (≥60g daily), women (≥40g daily)

Fig. S1a CVD-free life expectancy (LE free of CVD) and life expectancy with CVD (LE with CVD), in years at age 50 for men without CVD at baseline, RCPH (Denmark)

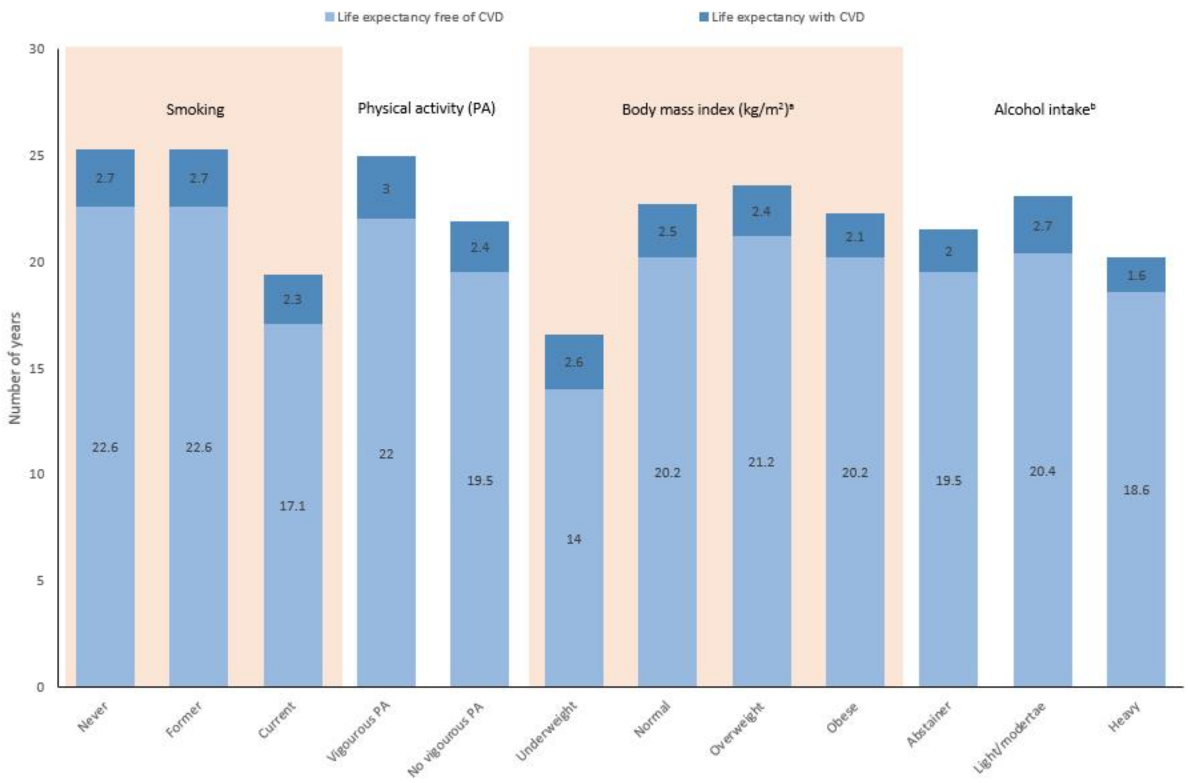

[a]Underweight = <18.5 kg/m^2; normal = ≥18.5 & <25; overweight = ≥25 & <30; obese = ≥30

[b]Light/moderate = >0g & <60g daily; Heavy = ≥60g daily

All corrected for age, history of diabetes, hypertension and total/HDL cholesterol ratio. Individually corrected for smoking status, physical activity, BMI and alcohol intake depending on model, i.e. Smoking corrected for physical activity, BMI and alcohol intake; BMI corrected for smoking status, physical activity, and alcohol intake

When using the repeated measures of covariates in the Markov model, the most recent available value for each measurement was used in the analysis

Fig. S1b CVD-free life expectancy (LE free of CVD) and life expectancy with CVD (LE with CVD), in years at age 50 for women without CVD at baseline, RCPH (Denmark)

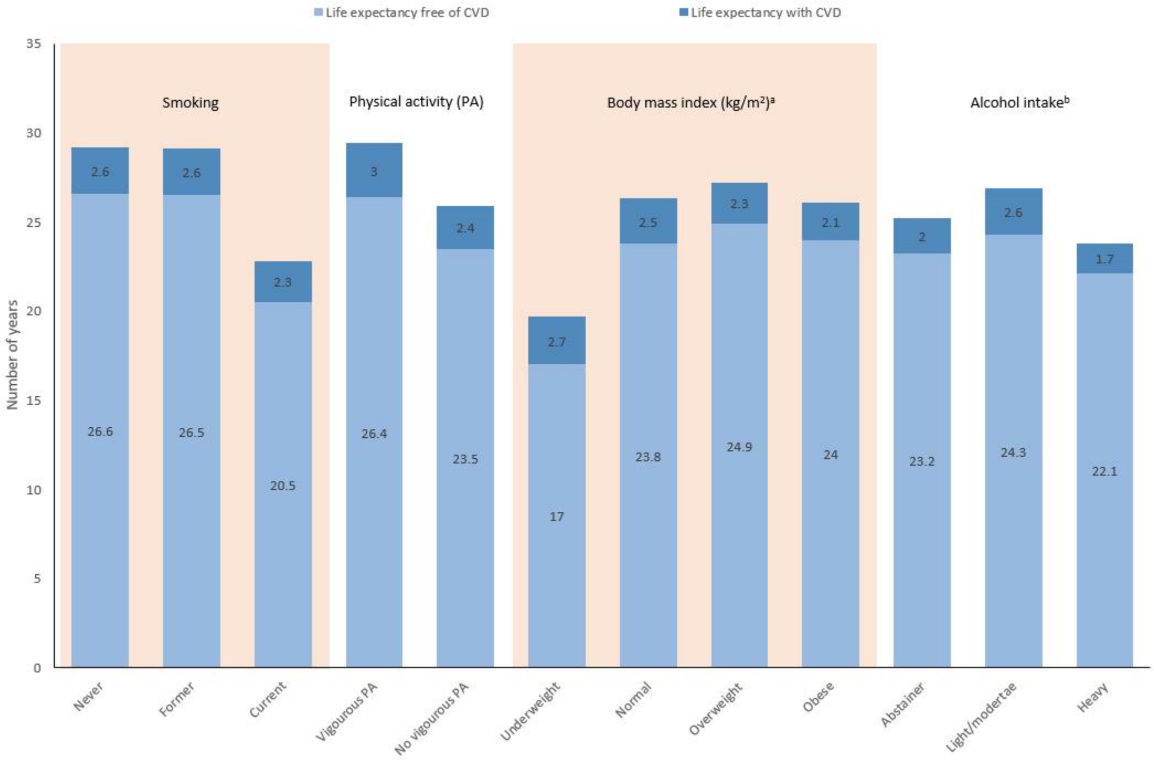

[a]Underweight = <18.5 kg/m2; normal = ≥18.5 & <25; overweight = ≥25 & <30; obese = ≥30

[b]Light/moderate = >0g & <40g daily; Heavy = ≥40g daily

All corrected for age, history of diabetes, hypertension and total/HDL cholesterol ratio. Individually corrected for smoking status, physical activity, BMI and alcohol intake depending on model, i.e. Smoking corrected for physical activity, BMI and alcohol intake; BMI corrected for smoking status, physical activity, and alcohol intake

When using the repeated measures of covariates in the Markov model, the most recent available value for each measurement was used in the analysis

Fig. S1c CVD-free life expectancy (LE free of CVD) and life expectancy with CVD (LE with CVD), in years at age 50 for men without CVD at baseline, ESTHER (Germany)

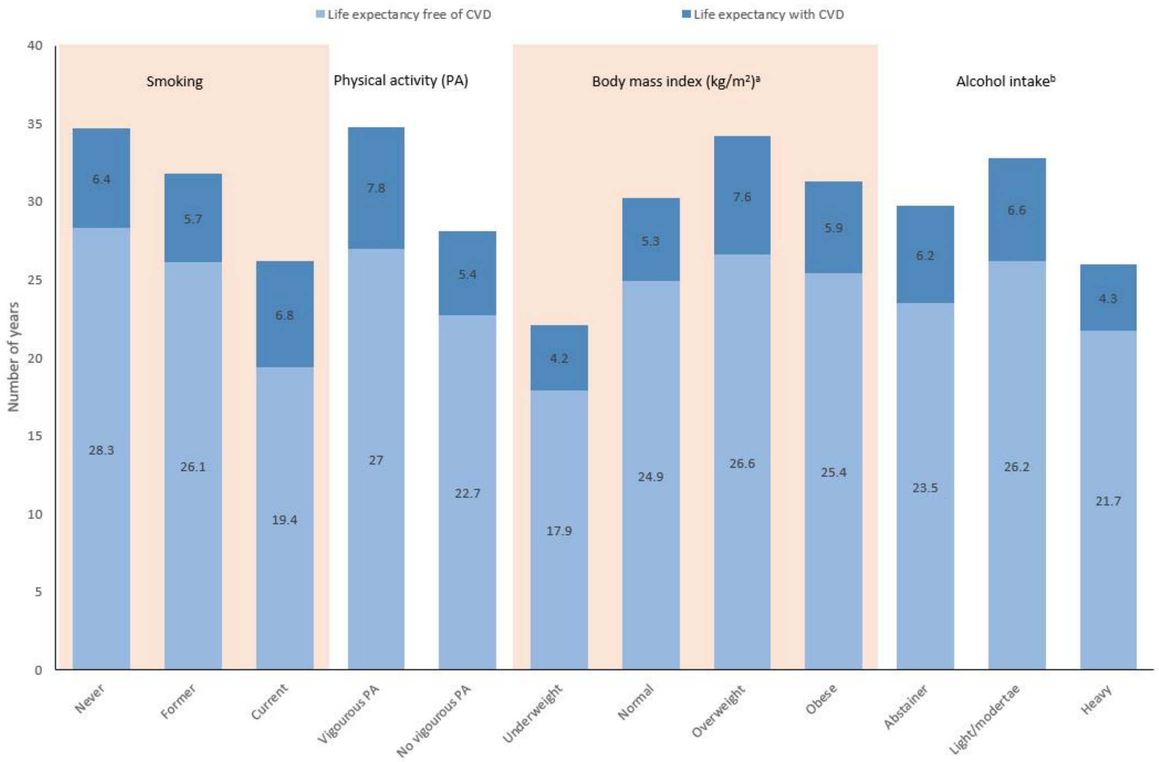

[a]Underweight = <18.5 kg/m2; normal = ≥18.5 & <25; overweight = ≥25 & <30; obese = ≥30

[b]Light/moderate = >0g & <60g daily; Heavy = ≥60g daily

All corrected for age, history of diabetes, hypertension and total/HDL cholesterol ratio. Individually corrected for smoking status, physical activity, BMI and alcohol intake depending on model, i.e. Smoking corrected for physical activity, BMI and alcohol intake; BMI corrected for smoking status, physical activity, and alcohol intake

When using the repeated measures of covariates in the Markov model, the most recent available value for each measurement was used in the analysis

Fig. S1d CVD-free life expectancy (LE free of CVD) and life expectancy with CVD (LE with CVD), in years at age 50 for women without CVD at baseline, ESTHER (Germany)

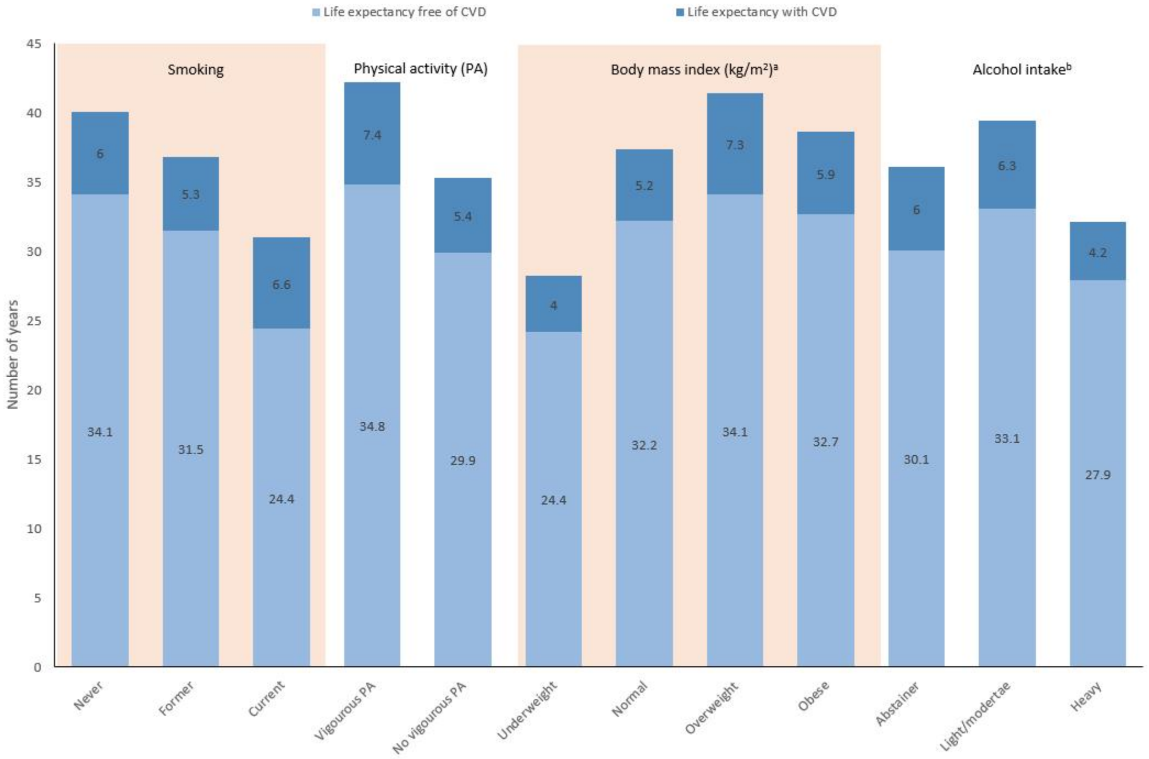

[a]Underweight = <18.5 kg/m2; normal = ≥18.5 & <25; overweight = ≥25 & <30; obese = ≥30

[b]Light/moderate = >0g & <40g daily; Heavy = ≥40g daily

All corrected for age, history of diabetes, hypertension and total/HDL cholesterol ratio. Individually corrected for smoking status, physical activity, BMI and alcohol intake depending on model, i.e. Smoking corrected for physical activity, BMI and alcohol intake; BMI corrected for smoking status, physical activity, and alcohol intake

When using the repeated measures of covariates in the Markov model, the most recent available value for each measurement was used in the analysis

Fig. S1e CVD-free life expectancy (LE free of CVD) and life expectancy with CVD (LE with CVD), in years at age 50 for men without CVD at baseline, Tromsø (Norway)

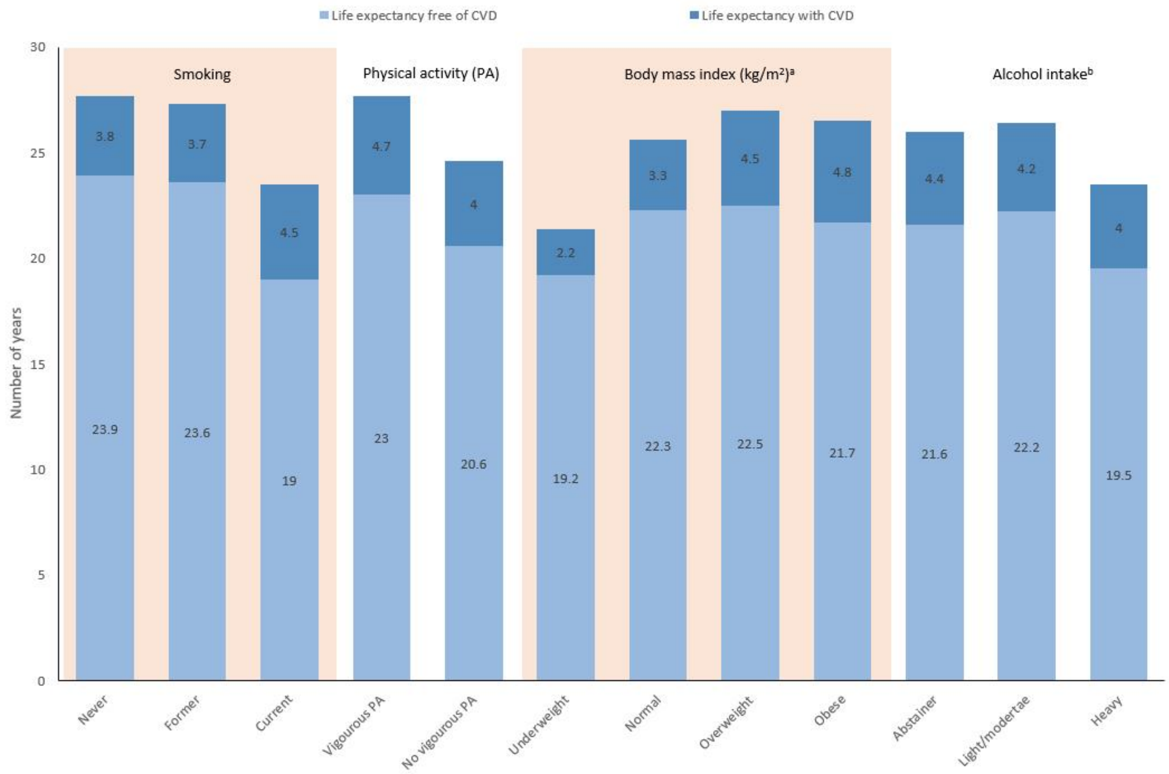

[a]Underweight = <18.5 kg/m2; normal = ≥18.5 & <25; overweight = ≥25 & <30; obese = ≥30

[b]Light/moderate = >0g & <60g daily; Heavy = ≥60g daily

All corrected for age, history of diabetes, hypertension and total/HDL cholesterol ratio. Individually corrected for smoking status, physical activity, BMI and alcohol intake depending on model, i.e. Smoking corrected for physical activity, BMI and alcohol intake; BMI corrected for smoking status, physical activity, and alcohol intake

When using the repeated measures of covariates in the Markov model, the most recent available value for each measurement was used in the analysis

Fig. S1f CVD-free life expectancy (LE free of CVD) and life expectancy with CVD (LE with CVD), in years at age 50 for women without CVD at baseline, Tromsø (Norway)

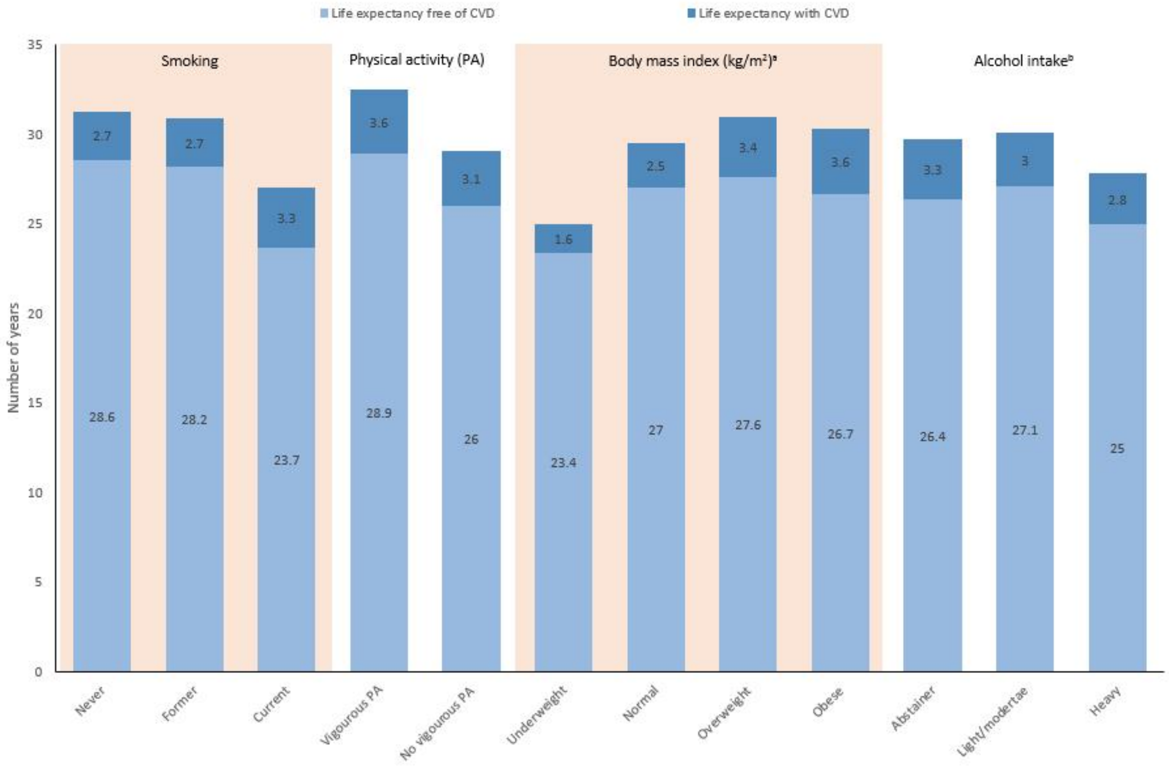

[a]Underweight = <18.5 kg/m2; normal = ≥18.5 & <25; overweight = ≥25 & <30; obese = ≥30

[b]Light/moderate = >0g & <40g daily; Heavy = ≥40g daily

All corrected for age, history of diabetes, hypertension and total/HDL cholesterol ratio. Individually corrected for smoking status, physical activity, BMI and alcohol intake depending on model, i.e. Smoking corrected for physical activity, BMI and alcohol intake; BMI corrected for smoking status, physical activity, and alcohol intake

When using the repeated measures of covariates in the Markov model, the most recent available value for each measurement was used in the analysis

References

1. Jackson CH. Multi-State Models for Panel Data: The msm Package for R. J Stat Softw. 2011;38:1–29.

2. Kalbfleisch J, Lawless J. The analysis of panel data under a Markov assumption. J Am Stat Assoc. 1985;80:863–71.

3. Rubin D. Multiple Imputation for Nonresponse in Surveys. New York: John Wiley & Sons; 1987.

4. Hout A van den, Jagger C, Matthews FE. Estimating Life Expectancy in Health and Ill Health by Using a Hidden Markov Model. J. R. Stat. Soc. Ser. C (Applied Stat. Wiley for the Royal Statistical Society; 2009;58:449–65.
